# Novel Hendra Virus Variant Detected by Sentinel Surveillance of Horses in Australia

**DOI:** 10.3201/eid2803.211245

**Published:** 2022-03

**Authors:** Edward J. Annand, Bethany A. Horsburgh, Kai Xu, Peter A. Reid, Ben Poole, Maximillian C. de Kantzow, Nicole Brown, Alison Tweedie, Michelle Michie, John D. Grewar, Anne E. Jackson, Nagendrakumar B. Singanallur, Karren M. Plain, Karan Kim, Mary Tachedjian, Brenda van der Heide, Sandra Crameri, David T. Williams, Cristy Secombe, Eric D. Laing, Spencer Sterling, Lianying Yan, Louise Jackson, Cheryl Jones, Raina K. Plowright, Alison J. Peel, Andrew C. Breed, Ibrahim Diallo, Navneet K. Dhand, Philip N. Britton, Christopher C. Broder, Ina Smith, John-Sebastian Eden

**Affiliations:** EquiEpiVet, Equine Veterinary and One Health Epidemiology, Aireys Inlet, Victoria, Australia (E.J. Annand);; Department of Agriculture, Water, and the Environment Epidemiology and One Health Section, Canberra (E.J. Annand, M.C. de Kantzow, A.C. Breed);; University of Sydney School of Veterinary Science and Institute for Infectious Diseases, Sydney, New South Wales, Australia (E.J. Annand, N. Brown, A. Tweedie, A.E. Jackson, K.M. Plain, N.K. Dhand);; CSIRO Health and Biosecurity Black Mountain Laboratories, Canberra, Australian Capital Territory, Australia (E.J. Annand, M. Michie, I. Smith);; Westmead Institute for Medical Research, Sydney (B.A. Horsburgh, K. Kim, J.-S. Eden);; University of Sydney School of Medicine, Sydney (B.A. Horsburgh, C. Jones, P.N. Britton, J.-S. Eden);; Ohio State University College of Veterinary Medicine, Columbus, Ohio, USA (K. Xu);; Private equine veterinary practice, Brisbane, Queensland, Australia (P.A. Reid);; Cooroora Veterinary Clinic, Cooroy, Queensland, Australia (B. Poole);; JData, Cape Town, South Africa (J.D. Grewar);; University of Pretoria, Pretoria, South Africa (J.D. Grewar);; CSIRO Australian Centre for Disease Preparedness, Geelong, Victoria, Australia (N.B. Singanallur, M. Tachedjian, B. van der Heide, S. Crameri, D.T. Williams);; Murdoch University School of Veterinary Medicine and The Animal Hospital, Murdoch, Western Australia, Australia (C. Secombe);; Uniformed Services University of the Health Sciences Microbiology and Immunology, Bethesda, Maryland, USA (E.D. Laing, S. Sterling, L. Yan, C.C. Broder);; Queensland Department of Agriculture and Fisheries Biosecurity Sciences Laboratory, Brisbane (L. Jackson, I. Diallo);; Children’s Hospital at Westmead, Infectious Diseases, Sydney (C. Jones, P.N. Britton);; Montana State University, Bozeman, Montana, USA (R.K. Plowright);; Griffith University Centre for Planetary Health and Food Security, Brisbane (A.J. Peel);; University of Queensland School of Veterinary Science, Gatton, Queensland, Australia (A.C. Breed)

**Keywords:** Hendra virus, zoonoses, viruses, Hendra henipavirus, viral encephalitis, meningitis/encephalitis, vasculitis, central nervous system, syndromic surveillance, syncytia, sentinel surveillance, One Health, horse diseases, sentinel species, RNA sequencing, whole-genome sequencing, PCR, transdisciplinary research

## Abstract

We identified and isolated a novel Hendra virus (HeV) variant not detected by routine testing from a horse in Queensland, Australia, that died from acute illness with signs consistent with HeV infection. Using whole-genome sequencing and phylogenetic analysis, we determined the variant had ≈83% nt identity with prototypic HeV. In silico and in vitro comparisons of the receptor-binding protein with prototypic HeV support that the human monoclonal antibody m102.4 used for postexposure prophylaxis and current equine vaccine will be effective against this variant. An updated quantitative PCR developed for routine surveillance resulted in subsequent case detection. Genetic sequence consistency with virus detected in grey-headed flying foxes suggests the variant circulates at least among this species. Studies are needed to determine infection kinetics, pathogenicity, reservoir-species associations, viral-host coevolution, and spillover dynamics for this virus. Surveillance and biosecurity practices should be updated to acknowledge HeV spillover risk across all regions frequented by flying foxes.

Highly pathogenic zoonotic Hendra virus (HeV) and Nipah virus (NiV) are prototypic members of the genus *Henipavirus*, family *Paramyxoviridae*, that have natural reservoirs in pteropodid flying foxes (*1*). These viruses exhibit wide mammalian host tropism, cause severe acute respiratory and encephalitic disease mediated by endothelial vasculitis, have high case-fatality rates, and cause chronic encephalitis among survivors (*2*–*4*). By March 2021, a total of 63 natural HeV spillovers had been recognized in horses in Australia, resulting in 105 horse deaths (*5*,*6*) and 4 deaths among 7 confirmed human cases (*7*). In southern Asia, NiV has caused zoonotic outbreaks with 70%–91% case-fatality rates, resulting in >700 human deaths (*8*–*10*). In response to the fatal disease threat posed by henipaviruses to humans and domestic animals, vaccines and postexposure prophylaxis (PEP) have been developed (*11*). A subunit vaccine, Equivac HeV (Zoetis, https://www.zoetis.com.au), based on the soluble recombinant G-attachment glycoprotein (receptor-binding protein [RBP]) of HeV (HeV-sG), that has been used for horses in Australia since 2012 (*12*). The human monoclonal antibody (mAb) m102.4 has been administered as emergency PEP in 16 human cases and has demonstrated safety, tolerability, intended pharmacokinetics, and no immunogenicity in a phase 1 trial (*13*). Combinations of cross-reactive humanized fusion (F) protein and RBP mAbs have also been described for clinical development as PEP (*14*–*16*), and a human vaccine using HeV-sG is now in phase 1 clinical trials (*17*).

Horses are the predominant species known to be infected with HeV by natural spillover from flying foxes; 2 canine (*18*) and all known human infections having resulted from close contact with infected horses. HeV transmission from *Pteropus* spp. (flying foxes) to horses is thought to occur primarily through contaminated urine (*19*). The spatial distribution of previously detected spillovers to horses and molecular HeV testing of flying fox urine suggested that transmission was predominantly from black flying foxes (BFF; *P. alecto*) and spectacled flying foxes (SFF; *P. conspicillatus*) (*19*,*20*). However, serologic testing has detected antibodies to HeV or related henipaviruses among all 4 flying fox species in Australia (*20*–*23*). Of note, seroprevalence of IgG targeting the HeV RBP has been reported in 43% of grey-headed flying foxes (GHFF; *P. poliocephalus*) in South Australia and Victoria (*22*) and 60% (169/284) in southeastern Queensland (*21*).

Australia hosts >1 million horses. Their grazing behavior, large respiratory tidal volume, and extensive highly vascularized upper respiratory epithelium may contribute to their vulnerability for HeV spillover (*23*). Detecting spillover to horses relies on attending veterinarians recognizing clinical manifestations consistent with HeV disease, sampling appropriately, and submitting samples for priority state laboratory testing (*24*). Passive surveillance using suspected disease testing is affected by a strong regional bias for areas where HeV has previously been detected and where domestic horse populations overlap with BFF distribution ranges, from eastern coastal Queensland to northern New South Wales (*25*). Testing for HeV is less commonly performed on horses with similar disease manifestations farther south in Australia because of a perception that spillover infection is less likely to occur in regions without BFF (*26*). Among >1,000 horses with manifestations consistent with HeV disease tested annually across regions of established risk, <1% are found to be positive (*25*,*27*). 

Routine testing for equine HeV infection as part of priority disease investigation is specific for the matrix (M) gene (*28*). Additional nucleoprotein (N) gene–specific testing (*29*) is limited to HeV-positive samples that undergo confirmatory testing (*30*) or in the minority (<7% nationally) of suspected equine HeV cases submitted directly to the national reference laboratory from states where spillover is considered less likely (*25*) and state testing is unavailable. This distinction is notable because it means that most horse-disease cases found negative for HeV are not investigated further, despite evidence that other viruses with potential spillover risk to horses, including novel related batborne paramyxoviruses, circulate in Australia (*27*,*31*–*35*). Likewise, animal health surveillance worldwide prioritizes targeted testing to exclude pathogens of established importance over open-ended diagnostic approaches, which are inherently more challenging to put in place and interpret.

Employing a transdisciplinary, interagency approach combining clinical-syndromic analysis and molecular and serologic testing, we explored the hypothesis that some severe viral disease–like manifestations in horses that are consistent with HeV, despite the horse testing negative, could be caused by undetected spillover of novel paramyxoviruses from flying foxes that potentially pose similar zoonotic risk. Here we report the identification of a previously unrecognized variant of HeV (HeV-var), circulation as a second genotypic lineage (HeV-g2), clinically indistinguishable from prototypic HeV infection, that resulted in severe neurologic and respiratory disease in a horse. 

## Materials and Methods

### Study Cohort

A biobank of diagnostic specimens collected in Queensland during 2015–2018 was developed from horses that underwent quantitative reverse transcription PCR (RT-PCR) testing but were negative for HeV (*28*). We recorded clinical, epidemiologic, and sample-related data, including vaccination status and perceived exposure to flying foxes (inconsistently reported by submitting veterinarians). All samples were archived at –80°C. We applied a decision algorithm based on systematic interpretation of pathologic basis and syndromic analysis of clinical disease descriptions to categorize each case by likelihood of infectious viral cause (Appendix Table). We plated samples (EDTA blood, serum, nasal swab, rectal swab) from cases assigned priority category 1 or 2 status, considered as having the highest likelihood of infectious cause, for serologic screening and high-throughput nucleic acid extraction using the MagMAX mirVANA and CORE pathogen kits (ThermoFisher, https://www.thermofisher.com).

### Pan-paramyxovirus RT-PCR Screening

We prepared cDNA from extracted RNA using Invitrogen SuperScript IV VILO Master Mix with ezDNase (ThermoFisher). A nested RT-PCR assay targeting the paramyxovirus L protein gene was adapted using primers developed elsewhere (*36*) and an AllTaq PCR Core kit (QIAGEN, https://www.qiagen.com). We identified amplicons corresponding to the expected size (584 bp) by gel electrophoresis before purification with AMPure XP (Beckman Coulter, https://www.beckmancoulter.com). To capture any weak detections, we also prepared pools by equal-volume mixing all PCR products across plated rows. We performed next-generation sequencing using an Illumina iSeq with the Nextera XT DNA library preparation kit (Illumina, https://www.illumina.com). For analysis, we assembled reads with MEGAHIT (*37*) before identifying them by comparison to GenBank entries using BLAST (*38*).

### HeV-var Whole-Genome Sequencing 

We subjected samples positive for HeV-var by paramyxovirus RT-PCR to meta-transcriptomic sequencing to determine the complete genome sequence and identify any co-infecting agents. RNA was reverse transcribed with Invitrogen SSIV VILO Master Mix (ThermoFisher) and FastSelect reagent (QIAGEN). We performed second-strand synthesis with Sequenase 2.0 (ThermoFisher) before DNA library preparation with Nextera XT (Illumina) and unique dual indexes. We performed sequencing on an Illumina NextSeq system to generate 100 million paired reads (75 bp) per library. 

### Assembly and Comparative Genomic and Phylogenetic Analyses

For genome assembly, we trimmed RNA sequencing reads and mapped them to a horse reference genome (GenBank GCA_002863925.1) using STAR aligner to remove host sequences. We assembled nonhost reads de novo with MEGAHIT (*37*) and compared them with the GenBank nucleotide and protein databases using blastn and blastx (*38*). We extracted the putative virus contig and remapped reads to this draft genome using bbmap version 37.98 (https://sourceforge.net/projects/bbmap) to examine sequence coverage and identify misaligned reads. We extracted, aligned, and annotated the majority consensus sequence by reference to the prototype HeV strain using Geneious Prime version 2021.1.1 (https://www.geneious.com) and submitted it to GenBank (accession no. MZ318101).

For classification, we aligned the paramyxovirus polymerase (L) protein sequence according to International Committee on Taxonomy of Viruses (ICTV) guidelines (*39*). We prepared alignments of partial nucleocapsid (N) and phosphoprotein (P) nucleotide sequences with known HeV strains from the GenBank database. Phylogenies were prepared using a maximum likelihood approach in MEGA X (https://www.megasoftware.net) according to the best-fit substitution model and 500 bootstrap replicates.

### Quantitative RT-PCR Development

We adapted quantitative RT-PCR targeting the HeV M gene (*28*) to target HeV-var. The duplex assay used the Applied Biosystems AgPath-ID One-Step RT-PCR kit (ThermoFisher) and distinguishes prototype and variant HeV strains. In brief, we combined 4 μL RNA with 10 μL 2× RT-PCR buffer, 0.8 μL 25× RT-PCR enzyme mix, 2 μL nuclease-free water, and 3.2 μL primer/probe mix (0.6 μL each primer, 0.3 μL each probe from 10 μmol stock; Table 1). We generated the reaction using 10 min at 50°C for cDNA synthesis, 10 min at 95°C for RT inactivation, and 50 cycles of 95°C for 15 s and 60°C for 30 s with FAM and HEX channels captured at the end of each cycle. As positive control, we synthesized gene fragments encoding a T7 promoter upstream of the partial M gene for both prototypic and variant HeV (Appendix Figure 1). We expressed RNA transcripts using the NEB HiScribe T7 High Yield RNA Synthesis kit (New England Biolabs, https://www.neb.com).

**Table 1:** Oligonucleotides used for duplex quantitative reverse transcription PCR targeting the matrix gene of novel Hendra virus variant from horse in Australia

Virus	Name	Sequence, 5′ → 3′*	Reference
Prototype	Mr_fwd_1	CTTCGACAAAGACGGAACCAA	(*34*)
	Mr_rev_1	CCAGCTCGTCGGACAAAATT	
	Mr_prb_1	FAM-TGGCATCTT-ZEN-TCATGCTCCATCTCGG-IABk	
Variant	Mv_fwd_1	TCTCGACAAGGACGGAGCTAA	Referent
	Mv_rev_1	CCGGCTCGTCGAACAAAATT	
	Mv_prb_1	HEX-TGGCATCCT-ZEN-TCATGCTTCACCTTGG-IABk	

*FAM and HEX 5′ reporter dyes were combined with ZEN Internal Quencher and the 3′ quencher Iowa Black, and supplied by Integrated DNA Technologies (https://www.idtdna.com).

### Virus Isolation, Confirmation, and Neutralization Studies

We attempted isolations in Vero cells (ATCC CCL-81) and primary kidney cells derived from black flying foxes (*40*). We confirmed them by cytopathogenic effect formation, quantitative RT-PCR, RNA sequencing, electron microscopy, and viral neutralization studies using HeV and isolated HeV-var mAb m102.4 (Appendix). 

### Serologic Analysis

We performed serologic analysis using multiplex microsphere immunoassays with a Luminex MAGPIX system (https://www.luminexcorp.com). We performed initial screening for IgG using an extensive panel of bacterial (*Leptospira*, *Brucella*) and viral antigens (paramyxovirus, filovirus, coronavirus, flavivirus, alphavirus) coupled to MagPlex beads (Bio-Rad, https://www.bio-rad.com) for multiplex screening. We added blood or serum diluted 1:100 to the beads, with binding detected following the addition of a combination of biotinylated-protein-G and -A and streptavidin-R-phycoerythrin. We read median fluorescence intensity on the MAGPIX system (Luminex) targeting 100 beads per antigen and used a Bayesian latent class model to assess test performance and determine appropriate cutoffs for positive test classification (*32*). We also applied an IgM assay in which biotinylated equine IgM was used in place of biotinylated proteins A and G.

### In Silico Analysis of the RBP Homology and mAb Binding

We compared the translated protein sequence of the HeV-var RBP sequence with established x-ray crystallography-derived structures of the HeV RBP protein structure bound to mAb m102.4 (*41*) and to ephrin-B2 using SWISS-MODEL (https://swissmodel.expasy.org). We used the results to assess the ability of m102.4 to neutralize this variant and further establish the likelihood of antibodies produced by immunization with the HeV vaccine being protective against this variant.

## Results

### Case Report

In September 2015, veterinary care was sought for a 12-year-old Arabian gelding in southeastern Queensland for severe disease consistent with HeV infection. The horse had always resided on the same property. Disease onset was acute; rapid deterioration occurred over 24 hours. Clinical assessment determined depressed (obtunded) demeanor, darkened red-to-purple change of the gingival mucous membranes with darker periapical line and prolonged capillary refill time, tachycardia (heart rate 75 beats/min), tachypnoea (60 breaths/min), normal rectal temperature (38.0°C), muscle fasciculations, head pressing, and collapse. 

HeV infection was suspected by the attending veterinarian, who had previously managed a confirmed case, on the basis of consistency with clinical disease manifestations and perception of plausible flying fox exposure. A nearby roost was known to host BFFs, GHFFs, and little red flying foxes (LRFF) of population sizes that varied seasonally and annually (*42*). Because of its moribund condition, the horse was humanely killed. We obtained postmortem nasal, oral, and rectal swab samples and combined them in 50 mL of sterile saline; we collected blood in an EDTA tube. Pooled swabs and blood samples were submitted to the Queensland Biosecurity Sciences Laboratory (Coopers Plains, Queensland, Australia) for priority HeV testing. Quantitative RT-PCR testing targeting the M gene did not detect viral RNA and ELISA testing did not detect HeV RBP IgG (*28*,*43*).

### Identification of Novel HeV-var

**Figure 1:**
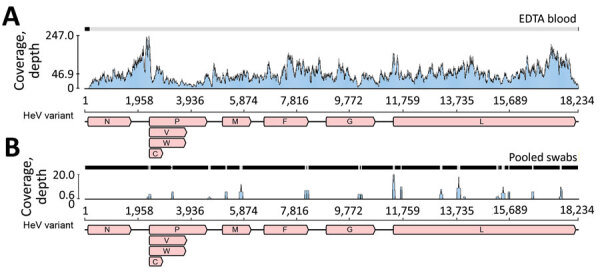
Sequence coverage of novel HeV variant from horse in Australia. The RNA sequencing reads were mapped to the novel HeV variant genome to examine coverage across the genome and depth for EDTA blood (A) and pooled swab samples (B). The x-axis shows the genome position with genes annotated and the y-axis shows the sequence read coverage (depth). Mean coverage depths were 46.9 for EDTA blood and 0.6 for pooled swab samples. V,W, and C indicate variably transcribed nonstructural proteins. F, fusion; G, glycoprotein; HeV, Hendra virus; M, matrix protein; N, nucleoprotein; P, phosphoprotein.

Given the high assigned likelihood of a zoonotic infectious cause (Appendix Table), we screened both the EDTA blood and pooled swab samples using pan-paramyxovirus RT-PCR (*36*). This identified the partial polymerase sequence of a novel paramyxovirus, most closely related to HeV (≈89% nt identity). Deep sequencing (mean coverage depth: 46.9×) of blood RNA generated the near–full-length genome of a novel HeV (Figure 1, panel A). The virus was less abundant in the pooled swab sample; mean coverage depth was 0.6× reads, spanning only 9.9% of the genome (Figure 1, panel B). No other viruses were present in either sample, and other microbial reads assembled were from common microflora, including *Staphylococcus aureus* and *Aeromonas*, *Veillonella*, *Pseudarthrobacter*, *Streptococcus*, *Acinetobacter*, and *Psychrobacter* spp.

### Confirmation of HeV Infection

**Figure 2:**
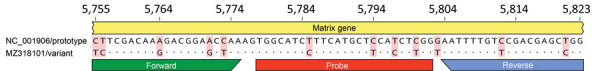
Genomic variation in the Hendra virus (HeV) matrix gene assay primer/probe binding sites for novel HeV variant from horse in Australia. The genomic region targeted by the commonly used HeV matrix gene quantitative RT-PCR assay (*28*) was aligned and compared for the prototype and variant HeV strains. The genomic positions relative to the prototype strain (GenBank accession no. NC_001906) are shown at the top. Primers (forward and reverse) and probe binding sites are indicated by the colored bars. Mismatches between the sequences are highlighted with red shading; dots indicate identical bases.

**Figure 3:**
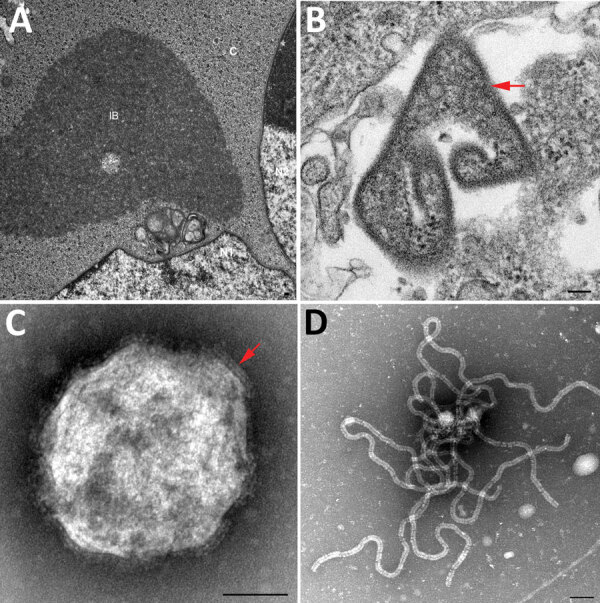
Transmission electron micrographs of Vero cells inoculated with the EDTA blood sample in study of novel Hendra virus variant from horse in Australia. A) Thin section showing inclusion body (IB) within the cytoplasm (C) of multinucleated (N1 and N2) syncytial cell. The nonmembrane bound IB consists of hollow nucleocapsids. B) Thin section showing virion (red arrow) with egress occurring at the plasma membrane. C) Negative contrast analysis shows a double-fringed envelope of the virion (red arrow). D) Negative contrast analysis shows strands of ribonucleic protein characteristic of the family *Paramyxoviridae*. Scale bars represent 100 nm.

A comparison of the primer and probe sequences used for the routine diagnostic PCR (*28*,*29*) revealed multiple mismatches in the binding sites, explaining the failure of routine surveillance to detect this variant (Figure 2). A quantitative RT-PCR assay was designed to detect both prototype and variant HeV strains in duplex (Table 1; Appendix Figures 1, 2), which amplified the templates of each virus with similarly high efficiency (>94%) and sensitivity, capable of detecting <100 copies of target RNA (Appendix Figure 2). The assay quantified results from the EDTA blood and pooled swabs samples, confirming RNA sequencing; the virus was more abundant in the EDTA blood (quantification cycle 26.87) than in the pooled swab samples (quantification cycle 30.67). We rescreened the priority cohort (864 samples from 350 cases in Queensland) using this novel assay but identified no additional cases. We successfully isolated virus from the EDTA blood sample of the case-animal in Vero cells. Electron microscopy of infected cells revealed cytoplasmic inclusion bodies (nucleocapsid aggregations; Figure 3, panel A) and enveloped viral-particle budding (Figure 3, panel B), consistent with HeV (Figure 3, panels A–D) (*44*).

Blood was tested for IgM and IgG against a panel of 33 antigens representative of bacterial and viral zoonoses (*32*,*45*), including RBPs of paramyxoviruses: HeV, NiV, Cedar henipavirus (CedV), Mojiang henipavirus (MojV), Ghana bat henipavirus (GhV), and Menangle Grove, and Yeppoon pararubulaviruses. We observed no notable reactions for this animal-case blood in either the IgG or IgM assays, indicating a lack of detectable antibodies consistent with acute viremia.

### Genomic Analysis of Novel HeV-var

**Figure 4:**
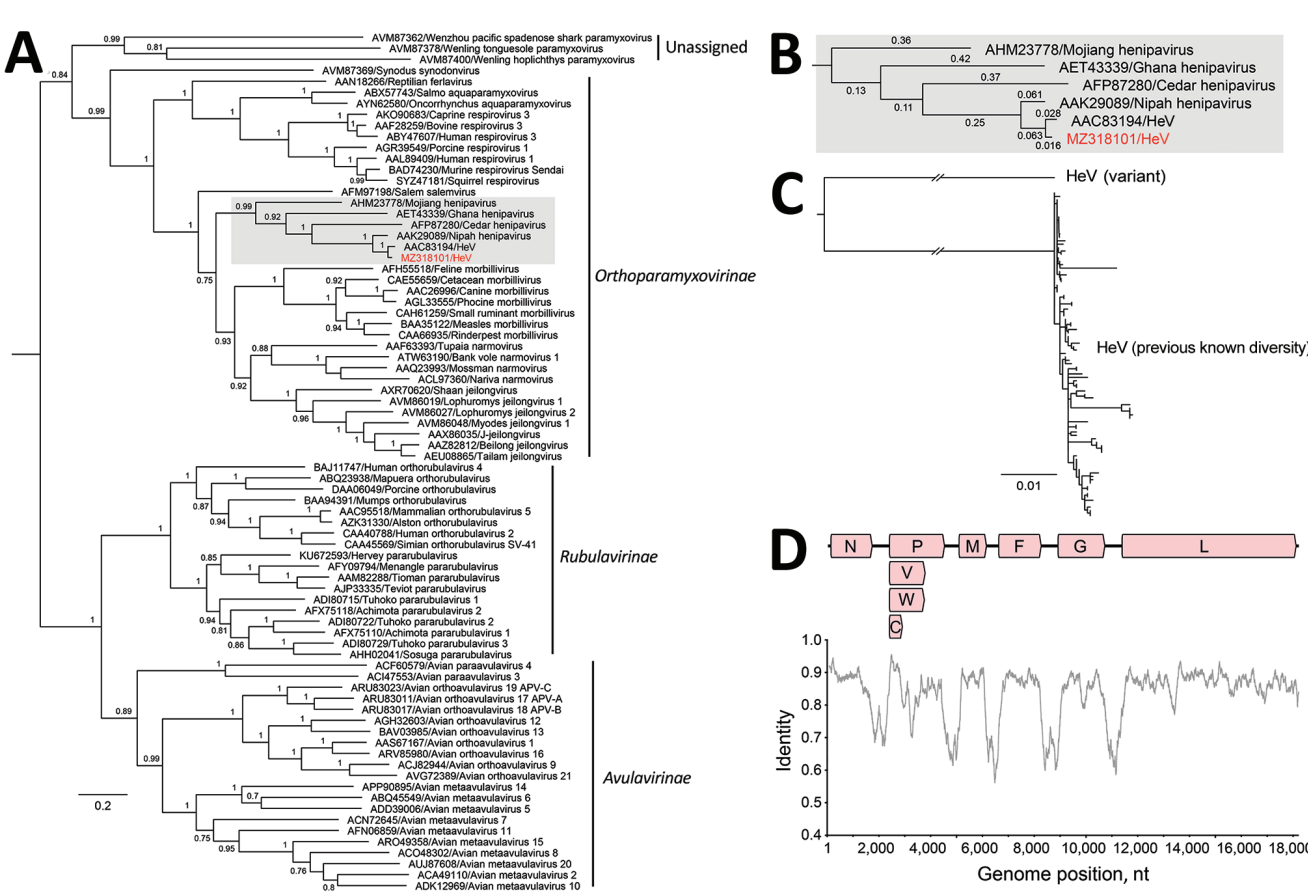
Phylogenomics of novel HeV variant from horse in Australia. A) Maximum-likelihood phylogeny of paramyxoviruses using complete L protein sequences. Gray shading indicates henipaviruses, and red text indicates the novel HeV variant, which groups with the prototypic HeV. Bootstrap support values as proportions of 500 replicates are shown at nodes; values <0.7 are hidden. Scale bar indicates substitutions per site. B) Enlarged gray area from panel A shows branch lengths for the henipavirus clade. The branch leading back to the common ancestor of all known HeVs and the novel HeV variant does not exceed 0.03; thus, they are considered variants of the same species. C) Maximum-likelihood phylogeny of partial N and P where deep branch lengths have been collapsed for visualization only to demonstrate that the variant is well outside the known diversity of HeV. Scale bar indicates substitutions per site. D) Nucleotide genomic similarity of the variant compared with the prototypic HeV strain. V,W, and C indicate variably transcribed nonstructural proteins. F, fusion; G, glycoprotein; HeV, Hendra virus; L, paramyxovirus polymerase; M, matrix protein; N, nucleoprotein; P, phosphoprotein.

We performed phylogenetic analyses of the novel HeV-var with other known paramyxoviruses (Figure 4, panels A–C). Comparison of the nucleotide similarity of the novel HeV-var to the HeV prototype strain (GenBank accession no. NC_001906) revealed an 83.5% pairwise identity across the genome (Figure 4, panel D). The L protein phylogeny revealed that the branch lengths of prototype and variant HeV to their common node did not exceed 0.03 substitutions/site (Figure 4, panels A, B). Therefore, the viruses were considered to be of the same species according to ICTV criteria (*39*). However, this HeV-var is clearly well outside known HeV diversity (Figure 4, panel C).

After this finding, comparison with a partial novel henipavirus M gene sequence derived from a GHFF from South Australia in 2013 (*46*) revealed 99% similarity to this HeV-var. This, along with additional subsequent flying fox detections (*47*), suggests that this HeV-var represents a previously undescribed lineage (HeV-g2), with reservoir-host infection across at least the range of this flying fox species.

### Analysis of the RBP

**Figure 5:**
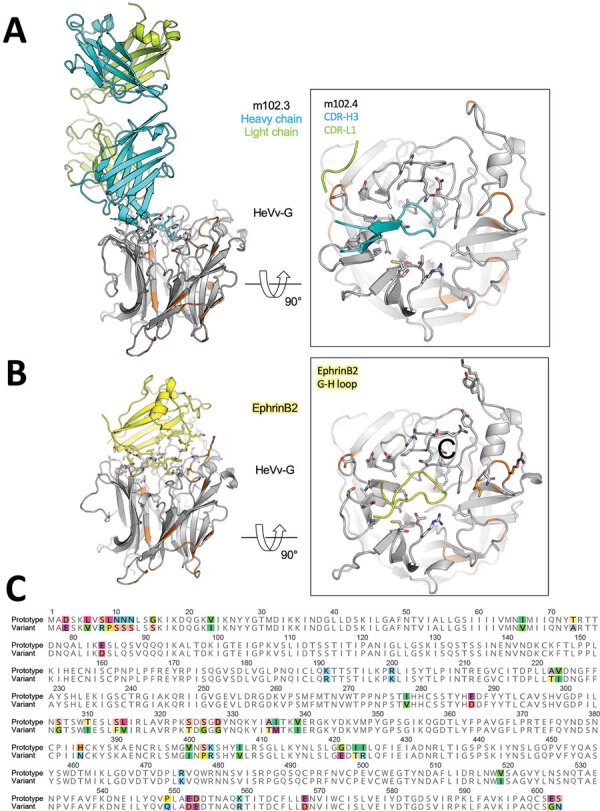
In silico modeling of HeV) from a horse in Australia. A, B) The translated protein sequence encoded by the HeV-var variant G gene was modeled using the known protein structure of the prototype virus bound to the human mAb m102.4 (A) and the receptor ephrinB2 (B). Side views (at left) of the interactions between the HeV G protein and the 2 binding partners highlight key binding residues (as sticks) and the variant positions (orange) relative to the m102.4 (heavy chain in teal and light chain in green) and ephrinB2 (in yellow). Zoomed top views (at right) of the HeV G and m102.4/ephrinB2 binding interface highlight specific interactions by the complementarity-determining regions of the mAb and G-H loop of the receptor ephrinB2. These data show that variable positions do not occur at critical epitopes at the HeV G and m102.4 binding interface and have very minor effect on the receptor ephrinB2 binding. C) Alignment of the prototypic and variant HeV strain G proteins. Variable positions are highlighted in color. F, fusion; G, glycoprotein; HeV, Hendra virus; mAb, monoclonal antibody.

Genomic sequencing showed greatest variability in the noncoding regions with mean pairwise genome identity higher (86.9%) across coding regions (Figure 4, panel D). At the protein level, this HeV-var shared 82.3%–95.7% (mean 92.5%) aa identity to the HeV prototype (Table 2). Of note, the HeV-var RBP shared 92.7% aa identity with prototypic HeV. Modeling of the novel HeV-var RBP structure based on the translated protein sequence using the x-ray crystal structure of the prototypic HeV RBP published elsewhere (*40*) supports that the epitopes for binding ephrin-B2 receptor and mAb m102.4 remain functionally unchanged because of consistency between key residues (Figure 5). Indeed, mAb m102.4 neutralization assays revealed equivalent neutralization potency of m102.4 (2.3 μg/mL of m102.4 neutralized 30 median tissue culture infectious dose of HeV-var and 4.6 μg/mL of m102.4 neutralized 300 median tissue culture infectious dose of HeV).

**Table 2:** Protein lengths of novel Hendra virus variant from horse in Australia and similarity to prototype strain*

Protein	Length, aa	Similarity, %
Nucleoprotein	532	96.6
Phosphoprotein	707	82.3
Matrix	352	95.7
Fusion	546	95.4
Glycoprotein	603	92.5
Large	2,244	95.7

*Prototype strain: GenBank accession no. NC_001906.

## Discussion 

We describe use of an innovative, syndromic risk-based targeted active sentinel surveillance activity for diagnostic investigation, extending from routine priority disease investigations, to identify a consequential virus. Based on ICTV criteria (*39*), this HeV-var is a novel genotypic variant of HeV, not a new *Henipavirus* species, but it evaded detection by routine diagnostic testing for HeV because of genomic divergence. Our findings highlight the potential of sentinel surveillance through One Health interagency and transdisciplinary syndromic infectious disease research to improve detection of emerging pathogens. We also describe a new assay for laboratory diagnosis and surveillance of this virus among humans and animals.

Comparing the translated amino acid sequences of this HeV-var and prototypic HeV RBP in silico revealed no change in the mAb m102.4 or ephrin-B2 entry receptor binding sites. Similarly, we confirmed equivalent m102.4 neutralization in vitro for this HeV-var and HeV. As such, it is expected that current PEP using mAb m102.4 will also be effective against this HeV-var. We emphasize that although the HeV RBP shares only 79% aa identity with NiV RBP, the HeV-sG subunit vaccine provided 100% protection against lethal challenge with both HeV and NiV in animal models (*11*). The high similarity between this HeV-var and HeV RBP (92.5% aa identity), structural consistency of critical epitopes, and equivalent in vivo viral neutralization assays also support that current vaccination using the HEV RBP will elicit similarly protective antibodies against HeV-var.

The 99% similarity between HeV-var and a partial M-gene sequence detected in a GHFF from Adelaide in 2013 highlights that a greater diversity of HeV strains than previously recognized circulates among flying fox species in Australia and that this novel variant likely circulates as a relatively consistent sublineage (HeV-g2), at least across the range of GHFF. Subsequent identification of HeV-g2 in GHFF and LRFF from regions without previous molecular HeV detection further support this understanding (*47*). 

Our findings indicate the urgent need for prompt reassessment of HeV spillover risk for horses and handlers living in southern New South Wales, Victoria, and South Australia, where risk for HeV infection has been perceived substantially lower than that in regions within the range of BFF distribution. Our findings indicate a need to update current molecular assays, which are not expected to distinguish between HeV and HeV-var (HeV-g2) and increase surveillance testing in horses and screening of flying foxes for HeV-g2 in these areas. These might further resolve the previously reported anomaly of high seropositivity despite low HeV detection within these species reported elsewhere (*20*–*22*).

Despite relatively high genetic divergence, the phenotypic similarity of this variant to prototypic HeV, combined with the observed consistency of disease manifestations in horses, suggests that the 2 strains have equivalent pathogenicity and spillover potential. Further characterization of HeV genomic diversity and any host-species associations will increase our understanding of transmission dynamics as well as virus-host coevolution features, such as possible codivergence or founder effects. Indeed, as climate change and anthropogenic habitat loss alter the extent and nature of interspecies interactions, BFFs have rapidly expanded their range southward, increasing their overlap with GHFFs (*48*). Sampling multiple species across time and space should inform how this variant strain circulates within and among flying fox species. Clearly, biosecurity practices should be updated to acknowledge spillover risk in all regions frequented by any species of flying fox.

Passive disease surveillance and biosecurity risk management for emerging diseases relies on recognition of suspected disease cases by clinical veterinarians, who play crucial roles relevant to animal and human health (*24*). Sporadic incidence of HeV and rare occurrence of Australian bat lyssavirus, yet high zoonotic consequence of both and lack of pathognomonic disease signs, inherently challenge surveillance of horses in Australia for these viruses. Critical and timely human postexposure management relies on confirmed animal-case diagnosis yet missed cases are inevitable, resulting in unmanaged risk of fatal zoonotic disease. Veterinarians are challenged in performing disease recognition by simultaneous obligations to serve both animals and animal owners, manage biosecurity risks, and meet Workplace Health and Safety Act and Biosecurity Act requirements (*24*,*49*,*50*). Veterinary description of disease manifestations most consistent with HeV led us to prioritize this case in our research testing pathway. This research detection of HeV-var highlights potential for improving emerging infectious disease surveillance through extending veterinarian-initiated risk-based suspect significant disease investigation, by selecting cases of highest likelihood of related viral cause and employing parallel serology and molecular testing pathways constructed to suit the available sample types and target diseases of highest clinical, species, and geographic relevance. These strategies build on the existing strength of systematic interpretation of clinical and field observations made by clinical veterinarians as part of existing submission and biosecurity protocols. This example serves as proof-of-concept for other disease contexts, highlighting the benefit of integrated transdisciplinary inquiry-based research approaches with routine biosecurity operations. Indeed, in October 2021, a fatal horse-case of HeV-g2 infection near Newcastle, New South Wales, was detected through an updated quantitative RT-PCR incorporated into routine priority disease testing.

Acknowledging the limitations of this single case, which lacked tissue for histopathology and immunohistochemistry, it is nonetheless appropriate that this HeV-var (HeV-g2) be considered equally pathogenic to prototype HeV based on coherent and consistent clinical signs of disease and pathology, evidence of viraemia, the phylogenetic analysis indicating that the variant belongs to the HeV species, and the modeling of the interactions of the functional RBP domain to the virus entry ephrin-B2 receptor. Moreover, this case fits the case definition for HeV infection in Australia’s AUSVETPLAN, which is that an animal tests positive to HeV using >1 of PCR, virus isolation, or immunohistochemistry (*50*). 

Updated PCR diagnostics suitable for routine priority detection of this HeV-var (HeV-g2) have been developed and are now used in many animal and human health laboratories in Australia. These findings demonstrate the limitation of exclusion-based testing for emerging zoonoses and a gap in our understanding of how frequently detection of known zoonoses across a broad range of systems are missed because of the diagnostic tools used. Further investigations to determine the prevalence of HeV-g2 circulation among and excretion from all flying fox species in Australia should be prioritized. The risk of zoonotic HeV disease in horses and human contacts should be interpreted across all regions frequented by all species of flying foxes, particularly those areas previously considered to be at low risk for HeV spillover. 

Appendix Additional information on novel Hendra virus variant detected by sentinel surveillance of horses in Australia.
