# Supplementary material for: Novel Hendra Virus Variant Detected by Sentinel Surveillance of Horses in Australia
Source: Emerg Infect Dis. 2022 Mar;28(3):693–704. doi: 10.3201/eid2803.211245 (PMC8888208; doi:10.3201/eid2803.211245)
Supplement: Appendix — Additional information on novel Hendra virus variant detected by sentinel surveillance of horses in Australia. [file 21-1245-Techapp-s1.pdf]

Article DOI: https://doi.org/10.3201/eid2803.211245

# Novel Hendra Virus Variant Detected by Sentinel Surveillance of Horses in Australia

## Appendix

## Supplementary Methods

### Viral Isolation

Positive case samples for the novel HeV were sent to the Australian Centre for Disease Preparedness (ACDP), a World Organization for Animal Health Reference Laboratory for Hendra and Nipah virus diseases, in line with established national arrangements for confirmatory testing of notifiable diseases of animals. Virus isolation was attempted in Vero cells (ATCC CCL-81) and primary kidney cells derived from *Pteropus alecto* (PaKi; *39*) on whole blood and pooled nasal, oral and rectal swab samples. Vero cells were grown at 37°C in EMEM (ThermoFisher; https://www.thermofisher.com) containing 10% fetal calf serum (FCS; ThermoFisher), supplemented with 1% v/v L-glutamine, 10 mM HEPES, 0.25% v/v penicillin–streptomycin and 0.5% v/v amphotericin B (Sigma-Aldrich; https://www.sigmaaldrich.com). PaKi cells were cultured in DMEM/F-12 media (ThermoFisher) with 5% FCS and supplemented with 1% v/v L-glutamine, 10 mM HEPES, 0.25% v/v penicillin–streptomycin and 0.5% v/v amphotericin B (Sigma-Aldrich).

For virus isolation, washed monolayers of cells were inoculated with 500 µL of whole blood diluted 1:5 in culture media or 500 µL of pooled swab sample prefiltered (0.45-µm cellulose acetate) to remove bacteria and any residual solid particles. Inoculum was removed after 45 min and cell monolayers were washed with phosphate-buffered saline, then overlaid with culture media containing 1% (v/v) FCS. Flasks were incubated at 37°C for 6–7 days and regularly monitored for cytopathic effect by light microscopy. Cells were then frozen, thawed and the cell suspension clarified by centrifugation (1000*g* at 4°C). Supernatant (500 µL) was then passaged onto fresh cell monolayers. A maximum of three passages per sample were

performed on each cell line. Final pass samples were tested by qRT-PCR to detect the presence of replicating HeV genome.

#### Electron Microscopy

For negative contrast EM, the clarified supernatant from Vero cell cultures, infected with HeV-var, were inactivated with 4% formaldehyde overnight. After adsorption of the inactivated supernatant onto formvar/carbon coated Cu400 grids, the preparation was then stained with Nano-W (Nano-probes) for 1 min. For thin section EM, the pelleted cells were fixed with modified Karnovsky fixative (4% formaldehyde and 2.5% glutaraldehyde in 0.1 M Sorensen's phosphate buffer) at 4$^{o}$C overnight. The pellet was rinsed in analogous buffer, fixed with 1% osmium tetroxide for 1 hr and dehydrated with a graded ethanol series prior to being embedded in Spurr's resin (ProSciTech; https://proscitech.com.au) according to manufacturer instructions. A Leica UC7 microtome was used to produce ultrathin sections, which were then stained in saturated uranyl acetate in 50% ethanol followed by lead citrate. Grids were examined and images acquired using a JEOL JEM-1400 transmission electron microscope at 120V.

#### Neutralization Assay

Serial dilutions of the mAb m102.4 and Hendra virus (isolate Hendra virus/Australia/Horse/2008/Redlands) or the HeV-var (Hendra-var/Australia/Horse/2015/Gympie) diluted to contain 100 $TCID_{50}$/well were incubated for 45–60 min at 37°C in a 96 well plate. A suspension of Vero cells was added to every well at a concentration of $4 \times 10^5$ cells per mL. Positive and negative serum controls and virus-only controls were included. Plates were incubated in a humid atmosphere containing 5% $CO_2$. Cells were examined after 3 days under an inverted microscope for cytopathic effect.

**Appendix Table.** Infectious disease prioritization categories (with examples) used in this study to identify Hendra-negative equine disease cases with highest likelihood of similar undiagnosed viral cause from larger cohort for further investigation

| Infectious disease priority | Description | Example |
|---|---|---|
| Category 1<br>Highest infectious disease suspect | Case features 'pyrexia' or 'abnormal mucous membranes AND one or more other clinical signs related to infectious disease OR the presence of either change AND 'epidemiological observation indicative of infectious cause' based on temporal and/or spatial relationship to similar disease cases | Pyrexia with tachycardia and acute onset respiratory consolidation and/or secretions. Pyrexia and neurological symptoms. Pyrexia and 'injected/congested' mucous membranes. 'Congested/injected mucous membranes' with acute severe respiratory dysfunction. Clustering of similar cases on same or neighboring properties |
| Category 2<br>High infectious disease suspect | Pyrexia OR other clinical signs associated with infectious disease of interest | Acute onset abnormal respiratory secretions. Fever of unknown origin. Colic with the presence of neurological symptoms |
| Category 3<br>Moderate infectious disease suspect | Clinical signs may be associated equally with infectious and noninfectious causes | Colic with the presence of dehydration and mucous membrane changes. Ataxia with the absence of pyrexia or known trauma |
| Category 4<br>Low infectious disease suspect | Non-infectious etiologies more common or most likely on differential diagnosis list, but infectious cause still possible | Ataxia following known traumatic event. Traumatic wounds following unusual behavioral event. Acute lethargy following chronic noninfectious disease condition |
| Category 5<br>No infectious disease suspect | No clinical signs of illness or no infectious cause considered likely | Traumatic wounds in the absence of underlying disease. Screening in unvaccinated horses to manage biosecurity risk prior to invasive procedures addressing non-infectious disease such as is a common requirement for dentistry or admission to equine hospitals in Australia |
| Category 6<br>Confirmed infectious disease | Other infectious disease confirmed via diagnostic testing | A case submitted for HeV testing, found negative and then testing positive for alternative known infectious disease such as ABLV, WNV, EHV or RRV* |

*ABLV, Australian bat lyssavirus; EHV, Equine herpes virus; HeV, Hendra virus; RRV, Ross River Virus; WNV, West Nile virus.

A

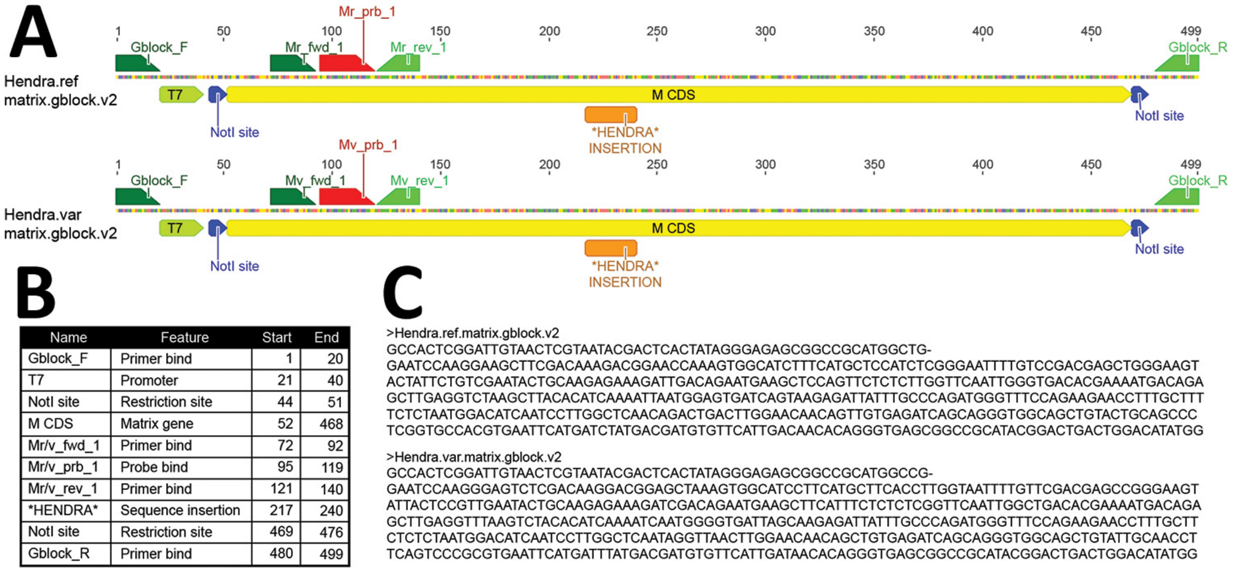


B

| Name | Feature | Start | End |
|---|---|---|---|
| Gblock_F | Primer bind | 1 | 20 |
| T7 | Promoter | 21 | 40 |
| NotI site | Restriction site | 44 | 51 |
| M CDS | Matrix gene | 52 | 468 |
| Mr/v_fwd_1 | Primer bind | 72 | 92 |
| Mr/v_prb_1 | Probe bind | 95 | 119 |
| Mr/v_rev_1 | Primer bind | 121 | 140 |
| *HENDRA* | Sequence insertion | 217 | 240 |
| NotI site | Restriction site | 469 | 476 |
| Gblock_R | Primer bind | 480 | 499 |

C

>Hendra.ref.matrix.gblock.v2
GCCACTCGGATTGTAACTCGTAATACGACTCACTATAGGGAGAGCGGCCGCATGGCTG-
GAATCCAAGGAAGCTTCGACAAAGACGGAACCAAAGTGGCATCTTTCATGCTCCATCTCGGGAATTTTGTCCGACGAGCTGGGAAGT
ACTATTCTGTCGAATACTGCAAGAGAAAGATTGACAGAATGAAGCTCCAGTTCTCTCTTGGTTCAATTGGGTGACACGAAAATGACAGA
GCTTGAGGTCTAAGCTTACACATCAAAATTAATGGAGTGATCAGTAAGAGATTATTTGCCCAGATGGGTTTCCAGAAGAACCTTTGCTTT
TCTCTAATGGACATCAATCCTTGGCTCAACAGACTGACTTGGAACAACAGTTGTGAGATCAGCAGGGTGGCAGCTGTACTGCAGCCC
TCGGTGCCACGTGAATTCATGATCTATGACGATGTGTTCATTGACAACACAGGGTGAGCGGCCGCATACGGACTGACTGGACATATGG

>Hendra.var.matrix.gblock.v2
GCCACTCGGATTGTAACTCGTAATACGACTCACTATAGGGAGAGCGGCCGCATGGCCG-
GAATCCAAGGGAGTCTCGACAAGGACGGAGCTAAAGTGGCATCCTTCATGCTTCACCTTGGTAATTTTGTTCGACGAGCCGGGAAGT
ATTACTCCGTTGAATACTGCAAGAGAAAGATCGACAGAATGAAGCTTCATTTCTCTCTCGGTTCAATTGGCTGACACGAAAATGACAGA
GCTTGAGGTTTAAGTCTACACATCAAAATCAATGGGGTGATTAGCAAGAGATTATTTGCCCAGATGGGTTTCCAGAAGAACCTTTGCTT
CTCTCTAATGGACATCAATCCTTGGCTCAATAGGTTAACTTGGAACAACAGCTGTGAGATCAGCAGGGTGGCAGCTGTATTGCAACCT
TCAGTCCCGCGTGAATTCATGATTTATGACGATGTGTTCATTGATAACACAGGGTGAGCGGCCGCATACGGACTGACTGGACATATGG

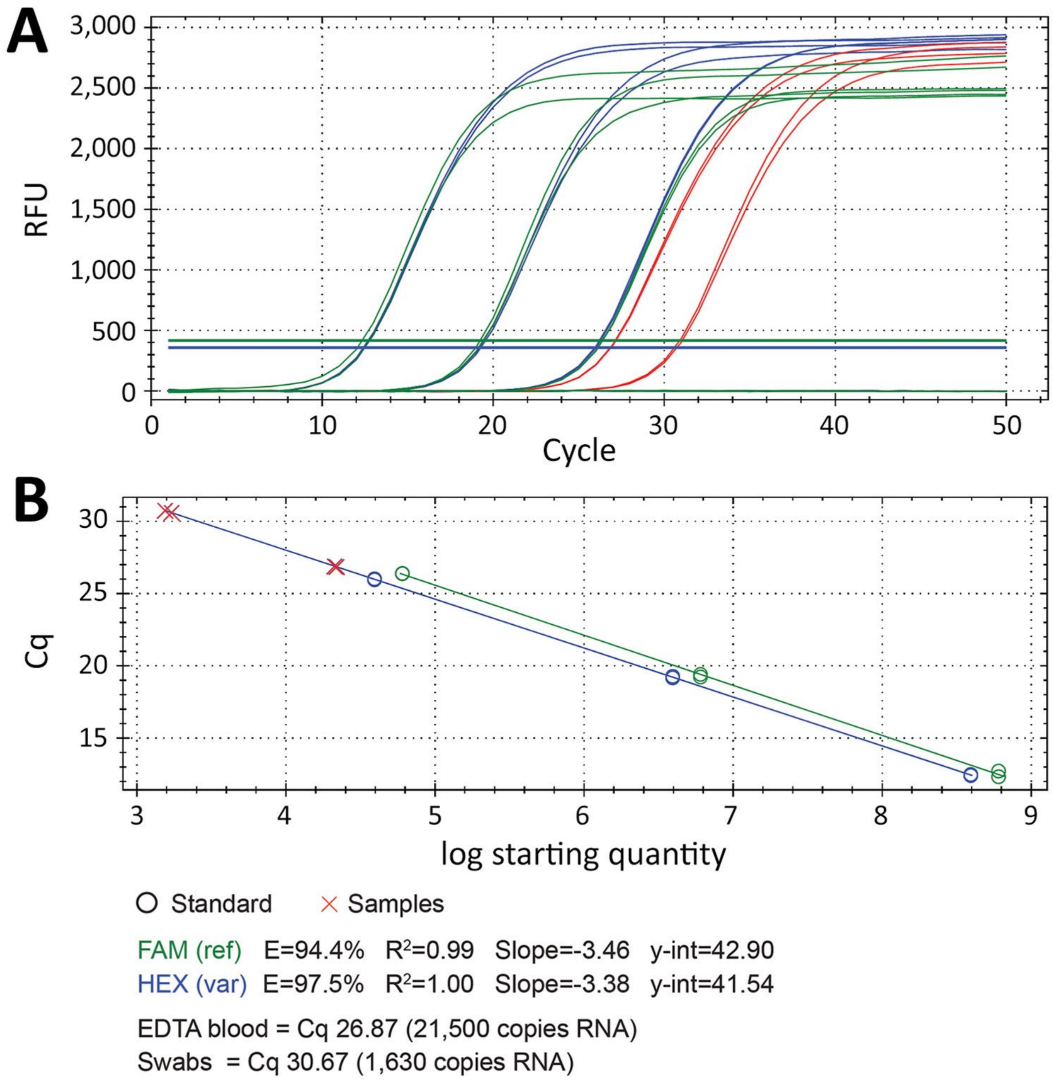


**Appendix Figure 2.** Quantitative reverse transcription PCR (qRT-PCR) performance and results for clinical samples. A) Amplification plot for duplex qRT-PCR assay reporting both reference (FAM [green]) and variant (HEX [blue]) HeV–strain channels with the synthetic RNA controls. Red traces show detections in the HEX channel for animal-case clinical EDTA blood and swab samples. B) Standard curve plot for duplex qRT-PCR assay reporting both FAM and HEX channels with the synthetic RNA controls.
